# Mechanisms of the IL-33/ST2 Signaling Axis in Regulating Bone Metabolism

**DOI:** 10.3390/biom16060811

**Published:** 2026-05-30

**Authors:** Libo Zhou, Zhongcheng Liu, Zirui Liu, Lei Wen, Bin Geng, Yayi Xia

**Affiliations:** 1 Department of Orthopaedics, Lanzhou University Second Hospital, Lanzhou University, Lanzhou 730030, China; 2 Orthopaedic Clinical Research Center of Gansu Province, Lanzhou 730030, China; 3 Intelligent Orthopaedic Industry Technology Center of Gansu Province, Lanzhou 730030, China

**Keywords:** IL-33, ST2, osteoporosis, bone immunology, diagnostic markers

## Abstract

Osteoporosis is a metabolic bone disorder characterized by reduced bone mass and deterioration of bone microarchitecture, resulting in increased skeletal fragility and an elevated risk of fracture. Its initiation and progression are closely linked to immune dysregulation and chronic inflammation. Interleukin-33 (IL-33), a key member of the interleukin-1 cytokine family, was initially identified as an alarmin that promotes type 2 immune responses. However, accumulating evidence has demonstrated that IL-33 plays a complex and pivotal role in the regulation of bone homeostasis, making it a molecule of considerable interest in osteoimmunology. This review aims to systematically summarize the mechanisms by which the IL-33/ST2 signaling axis regulates bone metabolism, further elucidate its multifaceted roles in primary and secondary osteoporosis, analyze its dual effects on bone protection and bone destruction, and evaluate its potential, as well as the associated challenges, as a diagnostic biomarker and therapeutic target.

## 1. Introduction

Osteoporosis (OP) is a common metabolic bone disorder characterized by reduced bone mass and increased skeletal fragility, resulting in an elevated risk of fracture [1]. The age-adjusted prevalence of OP among adults aged ≥50 years in the United States has been reported to be approximately 12.6%, and about 40% of women and 13% of men are expected to experience at least one osteoporotic fracture during their lifetime [2,3]. OP is generally classified into two major categories: primary and secondary osteoporosis [4]. Primary OP includes senile osteoporosis (SOP) and postmenopausal osteoporosis (PMOP) [5]. With progressive population aging worldwide, the incidence of PMOP is expected to increase substantially, posing a major challenge to public health. In contrast, secondary OP is commonly associated with conditions such as rheumatoid arthritis (RA), diabetes mellitus, and glucocorticoid exposure [6,7,8]. Bone homeostasis depends on a dynamic balance between osteoblast-mediated bone formation and osteoclast-driven bone resorption. However, age-related endocrine alterations, particularly estrogen deficiency in postmenopausal women, together with metabolic disturbances such as chronic hyperglycemia and obesity, suppress bone formation and enhance osteoclast activity, thereby contributing to bone loss and reduced bone strength [9,10].

In PMOP, increased osteoclast activity leads to a rise in bone resorption of up to 70%, whereas bone formation remains lower than or, at best, equivalent to bone resorption. Accordingly, PMOP is regarded as a high-turnover form of osteoporosis [11,12]. SOP affects both men and women, and its core mechanism lies in age-related cellular senescence, specifically a marked decline in the osteogenic differentiation capacity of bone marrow mesenchymal stem cells (BMSCs). Concurrently, senescent cells accumulate within the bone and secrete the senescence-associated secretory phenotype (SASP), leading to reduced bone formation and subsequent bone loss [13]. SOP is typically characterized as a low-turnover disease, with reduced bone resorption accompanied by an even more pronounced decline in bone formation [14]. RA-associated OP is essentially an inflammatory skeletal disorder driven primarily by autoimmune responses, in which activated T cells, especially T helper 17 (Th17) cells, B cells, and synovial fibroblasts produce pro-inflammatory cytokines such as TNF, IL-6, and IL-17 [15]. Diabetic osteoporosis (DOP) is characterized by impaired bone metabolism caused jointly by a hyperglycemic microenvironment and insulin resistance or deficiency. Hyperglycemia suppresses osteoblast differentiation and promotes osteoclast activity by inducing the accumulation of advanced glycation end products (AGEs) and enhancing oxidative stress. At the same time, insufficient insulin or insulin-like growth factor 1 (IGF-1) directly impairs the osteogenic function of osteoblasts [13,16]. Glucocorticoid-induced osteoporosis (GIOP) arises from the direct effects of exogenous glucocorticoids on bone cells and exhibits a dual detrimental pattern. In the early stage, glucocorticoids rapidly increase bone resorption by promoting osteoclast survival and activation through receptor activator of nuclear factor-κB ligand (RANKL). Meanwhile, long-term suppression of signaling pathways such as Wnt and bone morphogenetic protein (BMP) induces apoptosis and autophagy in osteoblasts and osteocytes, leading to persistent inhibition of bone formation and, ultimately, rapid bone loss and a marked increase in fracture risk [17,18,19].

Different forms of OP exhibit distinct core pathophysiological features. PMOP is characterized by excessive osteoclast activation triggered by estrogen deficiency; SOP is driven by the chronic inflammatory milieu created by senescent cell-derived SASP factors; RA-associated OP results from immune dysregulation-induced inflammatory damage; DOP is strongly associated with high glucose-induced metabolic and inflammatory disturbances; and GIOP reflects the combined suppression of bone formation and enhancement of bone resorption. Despite these differences, osteoimmune dysregulation is a common feature across the pathological processes of these forms of OP [20,21]. In PMOP, estrogen deficiency induces an imbalance in immune cell subsets, such as increased Th17/Treg and M1/M2 ratios [22,23,24]. In SOP, SASP contributes to the establishment of a chronic inflammatory microenvironment [25,26]. In RA, autoimmune effector cells secrete large amounts of pro-inflammatory mediators [27,28,29]. In DOP, hyperglycemia promotes immune activation, including M1 macrophage polarization [30,31]. In GIOP, glucocorticoids disrupt immune–bone signaling, for example by suppressing the anti-inflammatory functions of regulatory T cells (Tregs) and increasing the sensitivity of osteoclast precursors to RANKL [32,33]. Collectively, these mechanisms disrupt the dynamic balance between bone formation and bone resorption through disturbances in the bone–immune network. Importantly, the coupling between bone metabolism and immunity is not unidirectional but is mediated by reciprocal crosstalk between immune cells, including macrophages, T cells, and innate lymphoid cells, and bone cells, including osteoblasts, osteoclasts, and osteocytes. Specifically, cytokines secreted by immune cells directly regulate bone cell function, whereas bone cells can in turn modulate immune responses by releasing inflammatory mediators, thereby forming a complex osteoimmune regulatory network [34]. Disruption of this coupling mechanism is one of the shared drivers underlying bone loss across multiple forms of OP.

The homeostasis of the osteoimmune network depends on the precise regulation of multiple cytokines. Among these, the interleukin (IL) family plays a central role in communication between immune cells and bone cells [20,35]. IL-33, an important member of the IL-1 family, has attracted increasing attention because of its unique biological properties and diverse physiological and pathological functions since its identification and characterization [36]. On the one hand, IL-33 functions as an alarmin released in response to cellular or tissue damage, thereby alerting the immune system to injury [37]. On the other hand, as a conventional cytokine, it binds to interleukin-1 receptor-like 1 (IL1RL1/ST2) and interleukin-1 receptor accessory protein (IL-1RAcP) on the cell membrane, thereby activating downstream signaling pathways [38]. In addition, IL-33 can act as a nuclear factor that directly regulates gene expression [39]. Recent studies have shown that IL-33 is widely expressed in bone-related tissues and cells, including osteoblasts, osteocytes, osteoclasts, periodontal ligament cells, and immune cells [40,41,42]. Its expression is markedly upregulated under pathophysiological conditions such as inflammation, mechanical loading, and cellular injury, suggesting that it is deeply involved in regulation of the osteoimmune network. Accumulating evidence indicates that IL-33 directly or indirectly modulates osteoblast and osteoclast function by regulating osteoimmune pathways, including macrophage polarization and T-cell subset balance, thereby contributing to bone remodeling homeostasis [43,44,45]. 

**Figure 1:**
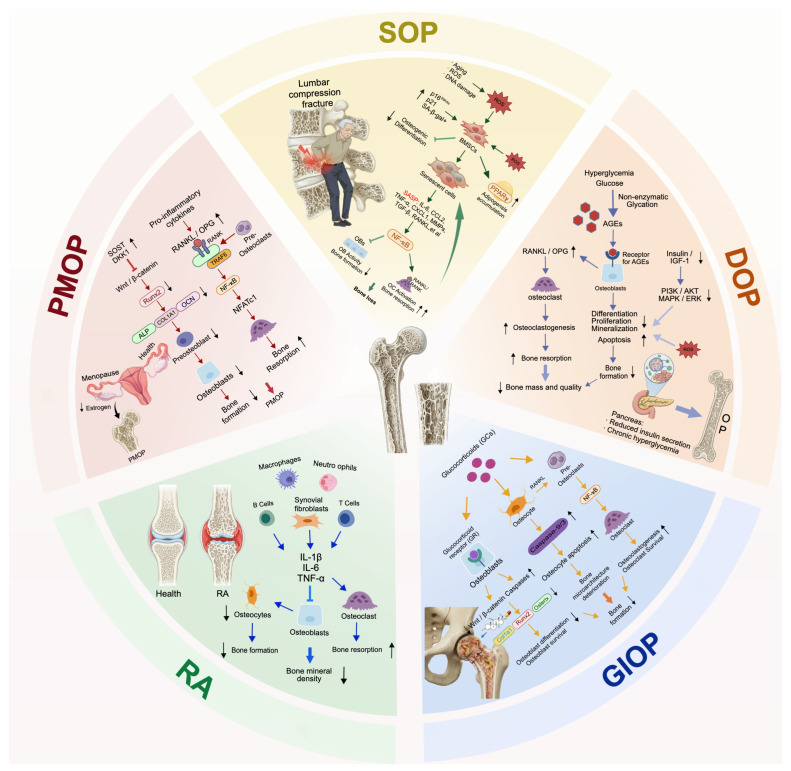
Molecular mechanisms underlying different types of osteoporosis.

However, despite substantial progress in research on the IL-33/ST2 axis, existing reviews still have important limitations. Sheng et al. [41] provided only a broad overview of the role of this axis in multiple systemic diseases, without an in-depth or systematic subtype-specific analysis of skeletal disorders. De Martinis et al. [46] focused exclusively on the IL-33/IL-31 axis in PMOP and did not address the full spectrum of osteoporosis subtypes or key skeletal conditions such as RA-related bone loss and osteonecrosis of the femoral head (ONFH). Wang et al. [47] limited their discussion to oral diseases and periodontal bone metabolism, without addressing systemic OP or osteoimmunology. To date, no review has systematically elucidated the differential expression patterns, subtype-specific regulatory mechanisms, and context-dependent functions of the IL-33/ST2 axis across diverse OP subtypes and related skeletal diseases. Therefore, this review comprehensively integrates current advances in PMOP, SOP, GIOP, DOP, RA-associated OP, and ONFH to address this gap. By systematically summarizing the biological characteristics, molecular mechanisms, clinical evidence, and ongoing controversies surrounding IL-33, this review highlights its subtype-specific roles in different skeletal diseases. Ultimately, we aim to provide a theoretical framework for exploring the IL-33/ST2 axis as a diagnostic biomarker and an immune-targeted therapeutic strategy for metabolic bone diseases (Figure 1).

## 2. The Molecular Biology of IL-33

### 2.1. Molecular Structure and Expression of IL-33

In 2005, Schmitz et al. [36] reported that the C-terminal region of IL-33 contains the characteristic β-trefoil fold of the IL-1 family, leading to its identification as the eleventh member of this cytokine family. The human *IL33* gene is located on chromosome 9p24.1, whereas the murine *IL33* gene is located on chromosome 19qC1 [48,49]. The gene contains a promoter region, exons, introns, and untranslated regions, spans approximately 4200 bp, and encodes a 270-amino-acid protein with a molecular weight of about 30.75 kDa. Structurally, IL-33 consists of three functional domains: an N-terminal nuclear domain (amino acids 1–65), a central domain (66–112), and a C-terminal IL-1-like domain (113–270) [50]. Due to the presence of a nuclear localization domain, IL-33 is predominantly localized in the nucleus under steady-state conditions. Upon cellular stress, injury, or death, IL-33 can be released into the cytoplasm and extracellular space, where it functions as a damage-associated molecular pattern (DAMP) or alarmin, alerting the immune system to tissue damage [51,52,53]. The precise mechanisms regulating IL-33 release remain incompletely understood, and recent studies suggest the existence of non-necrotic and cell death-independent release pathways [54,55,56,57]. Full-length IL-33 is biologically active upon release. In addition, proteolytic cleavage within the central domain can generate shorter mature forms with enhanced biological activity. In contrast, cleavage of IL-33 by caspase-3 and caspase-7 during apoptosis results in its inactivation [58,59].

### 2.2. IL-33 Receptors and Signaling Pathways

IL-33 forms a ternary complex with ST2 and IL-1RAcP, thereby activating intracellular signaling pathways [36,60]. ST2, also known as T1, IL1RL1, or DER4, is a member of the Toll-like/interleukin-1 receptor superfamily and is encoded by the IL1RL1 gene [61,62]. Two principal isoforms of ST2 have been described. One is the transmembrane form, termed ST2L, which mediates signal transduction. Upon complex formation with IL-33 and IL-1RAcP, ST2L recruits the adaptor protein myeloid differentiation primary response 88 (MyD88), together with interleukin-1 receptor-associated kinase 1 and 4 (IRAK1/4), leading to activation of tumor necrosis factor receptor-associated factor 6 (TRAF6) and downstream pathways, including nuclear factor κB (NF-κB), mitogen-activated protein kinase (MAPK), and phosphatidylinositol 3-kinase/protein kinase B (PI3K/Akt) signaling [61,63]. The other major isoform is the soluble form, sST2, which lacks a transmembrane domain and is released into the extracellular space. sST2 acts as a decoy receptor by sequestering IL-33 and thereby preventing signal transduction [64,65]. Prior to the discovery of IL-33, ST2 was considered an orphan receptor.

**Figure 2:**
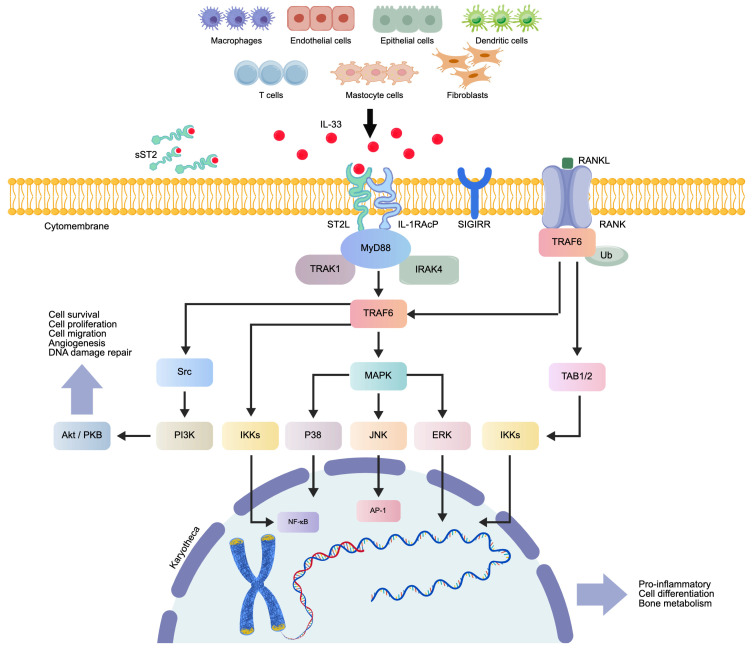
IL-33 binds to ST2L and IL-1RAcP, or to sST2.

Studies have shown that ST2 is expressed on the surface of various cell types, including T cells, mast cells (MCs), macrophages, dendritic cells, basophils, eosinophils, natural killer cells, and group 2 innate lymphoid cells (ILC2s) [66,67] (Figure 2).

### 2.3. The Multiple Biological Functions of IL-33

IL-33 is a key molecule that functions as both an alarmin and a cytokine. It broadly regulates innate and adaptive immune responses and exerts substantial effects on the activation, differentiation, and function of macrophages, natural killer cells, MCs, ILC2s, and multiple T and B cell subsets, thereby contributing to host defense and immune homeostasis [48,68,69,70]. In particular, IL-33 is a major driver of type 2 immune responses. It promotes Th2 cell polarization and induces the secretion of cytokines such as IL-4, IL-5, and IL-13 [71], regulates the activation and recruitment of eosinophils and basophils [72], and plays a leading role in Type 2 inflammation and allergy-related immune processes. IL-33 is also fundamentally involved in inflammatory responses, tissue repair, and metabolic disorders. As a DAMP, it rapidly responds to tissue damage, initiating and regulating both acute and chronic inflammation [68,73]. IL-33 contributes to the repair and regeneration of multiple organs, including the skin, skeletal muscle, heart, intestine, and kidney, by promoting reparative macrophage polarization and stimulating Tregs and ILC2s to secrete anti-inflammatory factors and tissue-repair mediators [74,75,76,77,78,79]. In metabolic diseases, IL-33 influences glucose uptake, glycolysis, cellular insulin sensitivity, and the transition of adipocytes between white and brown adipose phenotypes [80,81,82,83]. Furthermore, IL-33 regulates the local immune microenvironment during processes such as organ fibrosis and bone metabolism disorders, thereby maintaining tissue homeostasis and pathophysiological balance [41,43,84,85,86].

## 3. Core Molecular Mechanisms of IL-33 in Regulating Bone Metabolism

### 3.1. The Effects of IL-33 on Osteoclasts

IL-33 suppresses RANKL-induced osteoclastogenesis by modulating key transcriptional networks involved in osteoclast differentiation, which represents one of the core mechanisms underlying its bone-protective effects [87]. Osteoclast differentiation depends critically on the activation and amplification of nuclear factor of activated T cells, cytoplasmic 1 (NFATc1). As a master transcription factor for osteoclastogenesis, NFATc1 drives the expression of a series of osteoclast-specific genes, including TRAP, cathepsin K, and integrin β3, ultimately driving the formation of multinucleated mature osteoclasts [88,89,90,91]. Available studies have shown that IL-33 significantly downregulates NFATc1 expression and inhibits its nuclear translocation, thereby blocking osteoclast differentiation at an early stage [87].

At the molecular level, IL-33 does not merely inhibit NFATc1 directly, but also exerts negative regulatory effects by upregulating transcription factors that inhibit osteoclast differentiation, including B-lymphocyte-induced maturation protein 1 (Blimp1), interferon regulatory factor 8 (IRF-8), and v-maf musculoaponeurotic fibrosarcoma oncogene homolog B (MafB). These molecules function as endogenous negative regulators of osteoclastogenesis by directly repressing NFATc1 transcription or competitively interfering with the expression of downstream target genes. Consequently, the directed differentiation of bone marrow-derived monocytes/macrophages (BMMs) into the osteoclast lineage is impeded, leading to a decrease in the number and maturity of multinucleated osteoclasts [92,93]. These molecular mechanisms have been further validated by in vitro experiments. In an osteoclast differentiation system induced by RANKL and tumor necrosis factor-α (TNF-α), treatment with IL-33 (100 ng/mL) significantly reduced the number of TRAP-positive multinucleated giant cells and markedly suppressed the expression of osteoclast-specific functional genes [94]. Concurrently, under ST2 knockout or blockade conditions, the inhibitory effect of IL-33 on osteoclasts was markedly attenuated, suggesting that this effect is mediated by the IL-33/ST2 axis [95]. Collectively, these findings indicate that IL-33 efficiently inhibits osteoclast differentiation through a dual regulatory mechanism that upregulates anti-osteoclastogenic transcription factors and downregulates core pro-osteoclastogenic factors, thereby reducing bone resorption and maintaining bone mass homeostasis. In addition to directly inhibiting osteoclast differentiation, IL-33 can also accelerate apoptosis in mature osteoclasts, thereby further reducing the number of functional osteoclasts and exerting a bone-protective effect. Studies have confirmed that IL-33 significantly upregulates the expression levels of key pro-apoptotic proteins such as Bax, Fas, and FasL in osteoclasts, while simultaneously downregulating anti-apoptotic molecules such as Bcl-2. This disrupts the apoptotic balance within osteoclasts, activates the endogenous mitochondrial apoptosis pathway and the extrinsic death receptor pathway, and ultimately promotes rapid apoptosis of mature osteoclasts [40].

However, although numerous studies support an inhibitory effect of IL-33 on osteoclastogenesis, there is also evidence suggesting that, under certain conditions or through the mediation of specific cell types, IL-33 can indirectly promote osteoclast differentiation and activity. A study by Kim et al. [96] revealed that IL-33 can activate MCs, significantly upregulating the expression of various pro-inflammatory and osteoclastogenic factors, including TNF-α, IL-1β, IL-6, IL-17, and RANKL. Further studies found that IL-33-activated MCs can promote the differentiation of CD14^+^ monocytes into osteoclasts through direct cell–cell contact or the secretion of soluble factors. This finding suggests that in inflammatory bone diseases such as RA, IL-33 may indirectly promote bone resorption by activating MCs. However, whether IL-33 can directly promote human osteoclastogenesis remains controversial. Mun et al. [97] used magnetically purified adult peripheral blood CD14^+^ monocytes and reported that, in the absence of exogenous RANKL, IL-33 induced the differentiation of CD14^+^ monocytes into TRAP^+^ multinucleated cells via ST2, accompanied by activation of Syk/PLCγ2, MAPK, NF-κB, and NFATc1 signaling, ultimately leading to bone-resorptive function. In contrast, Eeles et al. [98], using an adult peripheral blood mononuclear cells (PBMC) adherence culture system, found that IL-33 induced dentine resorption pit formation in only a small subset of donors, and its effect was markedly weaker than that of RANKL. In addition, IL-33 showed no direct pro-osteoclastogenic effect on cord blood-derived CFU-GM progenitors or murine bone marrow-derived macrophages, and it failed to effectively induce NFATc1 in RAW264.7 cells, exhibiting only very weak osteoclastogenic activity. The discrepancy between these two studies may be attributable to several specific methodological factors, including differences in the starting cell populations (purified CD14^+^ monocytes vs. PBMC adherence cultures), inter-donor variation in ST2 expression and downstream signaling context, the presence of endogenous co-stimulatory factors in the culture system, differences in the source and dose of recombinant IL-33, and the criteria used to define functional osteoclasts. Current evidence thus suggests that any direct pro-osteoclastogenic effect of IL-33 is context-dependent rather than a universal biological feature. Future studies should employ matched cell sources, standardized IL-33 preparations, and rigorously defined culture systems, together with ST2 blockade, RANKL/OPG intervention, and bone resorption assays, to more definitively determine whether IL-33 truly exerts a direct pro-osteoclastogenic effect.

### 3.2. The Effects of IL-33 on Osteoblasts

As a pleiotropic cytokine, IL-33 exerts controversial effects on osteoblasts. Existing studies have revealed its complex role in regulating osteoblast differentiation and bone formation.

Saleh et al. [99] found that IL-33 promotes matrix mineralization in primary osteoblasts and reduces the mRNA levels of sclerostin (SOST), suggesting a role for IL-33 in supporting osteoblast function. Additionally, the study found that parathyroid hormone (PTH) and oncostatin M can upregulate IL-33 expression in osteoblasts, further supporting the potential positive role of IL-33 in osteogenesis. Furthermore, Qiu et al. [100] found that macrophages secrete IL-33 upon stimulation by black phosphorus. This cytokine achieves a temporal balance between early pro-inflammatory and late anti-inflammatory responses by regulating factors such as IL-6, TNF-α, IL-10, and IGF-1. Concurrently, IL-33 directly acts on BMSCs to upregulate the expression of osteogenesis-related genes, thereby promoting mineralization and calcium deposition. However, Saidi et al. [101] reported that although IL-33 mRNA is expressed in human osteoblasts, its receptor ST2L is not constitutively expressed in these cells, and IL-33 has no significant direct effect on osteoblast differentiation, proliferation, or the expression of genes related to bone formation. This study suggests that during normal bone remodeling, IL-33 may not directly regulate osteoblast function, and its role may be redundant with other factors. These discrepancies may be attributable to several factors. First, differences in cell origin. Osteoblasts from different species (e.g., mice vs. humans) or different tissue sources (e.g., primary cells vs. cell lines) may respond differently to IL-33. Second, the influence of the stage of differentiation. IL-33 may only play a role at specific stages of osteoblast differentiation, rather than being involved throughout the entire process. Furthermore, differences in the microenvironment should not be overlooked. Factors such as in vitro culture conditions, the presence of other cytokines (e.g., TNF-α and IL-1β), or mechanical stimulation may all influence the effects of IL-33. Finally, the expression level of ST2 on the surface of osteoblasts may determine their sensitivity to IL-33.

Although IL-33 may not directly regulate osteoblast differentiation, it can indirectly influence osteoclastogenesis by affecting the secretion of key bone remodeling factors by osteoblasts. Mine et al. [102] reported that IL-33 significantly upregulated RANKL mRNA and protein levels in MC3T3-E1 cells, but had no significant effect on the expression of osteoprotegerin (OPG). This effect depends on the ERK and p38 MAPK signaling pathways, rather than the JNK pathway. By upregulating the RANKL/OPG ratio, IL-33 may indirectly promote osteoclast differentiation and activity. Heckt et al. [103] further found that although IL-33 can induce osteoblasts to express the RANKL-encoding gene (Tnfsf11), unlike PTH, IL-33 does not promote the release of soluble RANKL. This suggests that IL-33 may participate in bone remodeling primarily by modulating membrane-bound RANKL expression, rather than by promoting its proteolytic processing.

### 3.3. Effects of IL-33 on Osteocytes

Following terminal differentiation of osteoblasts, a subset of these cells further differentiates into osteocytes. Research by Saleh et al. [99] suggests that IL-33 influences osteocyte function by reducing the expression levels of SOST derived from osteocytes. As SOST is a negative regulator of bone formation, IL-33–mediated downregulation of SOST may promote osteogenesis. Noguchi et al. [104] found in osteocyte-like MLO-Y4 cells that IL-33 can activate the NF-κB, JNK/AP-1, and p38 MAPK signaling pathways via the ST2L receptor, significantly upregulating IL-6 expression. Given that IL-6 is a key regulator of bone remodeling, this finding suggests that IL-33 may indirectly participate in bone metabolic regulation through its effects on osteocytes.

### 3.4. The Synergistic Regulatory Role of IL-33 in the Bone Immune Network

IL-33 indirectly inhibits osteoclastogenesis at the level of the immune microenvironment by regulating the function of local bone immune cells, reflecting the characteristics of osteoimmune coupling. As IL-33 is known to activate ILC2s, Omata et al. [105] exploited this property to expand ILC2s and further demonstrated that activated ILC2s directly inhibit osteoclast precursor differentiation via the STAT6 signaling pathway by secreting IL-4 and IL-13, thereby attenuating bone loss in a PMOP model. Furthermore, in arthritis models, ILC2s similarly inhibit the secretion of inflammatory factors such as IL-1β by macrophages via IL-4 and IL-13, thereby reducing local bone erosion [106]. On the other hand, IL-33 can effectively inhibit the polarization and activation of M1 macrophages, reduce the release of pro-inflammatory factors such as TNF-α and IL-1β, and mitigate alveolar bone destruction. It also promotes the transition of macrophages toward an anti-inflammatory M2 phenotype, thereby lowering local inflammation levels and indirectly maintaining bone metabolic balance [107]. Zaiss et al. [44] found that IL-33 exerts a protective effect by inhibiting bone resorption both in vivo and in vitro through direct action on osteoclast precursor cells, promoting their differentiation into alternatively activated macrophages (AAMs). Momiuchi et al. [108] further extended this concept from the perspective of immune cell interactions, finding that IL-33 stimulates ILC2s in the bone marrow, causing them to downregulate RANKL expression and produce granulocyte-macrophage colony-stimulating factor (GM-CSF) and IL-13. These factors promote the polarization of BMMs toward M2 macrophages rather than osteoclast differentiation, thereby inhibiting osteoclastogenesis. According to Zhang et al. [109], the IL-33/ST2 signaling pathway is one of the key mechanisms driving the expansion and functional activity of reparative Tregs in damaged tissues. In the skeletal system, Tregs can suppress osteoclastogenesis by secreting IL-10 and TGF-β, as well as through direct cell–cell contact, thereby maintaining bone homeostasis and promoting bone repair in pathological conditions such as arthritis. Similarly, Ünsal et al. [110] further noted in the context of periodontitis that IL-33 is a crucial factor in regulating Treg function. In chronic inflammatory diseases such as periodontitis, the balance between Tregs and Th17 cells determines the direction of disease progression. By enhancing Treg suppressive activity, IL-33 suppresses Th17-mediated inflammatory responses and osteoclast activation, thereby mitigating alveolar bone destruction.

**Figure 3:**
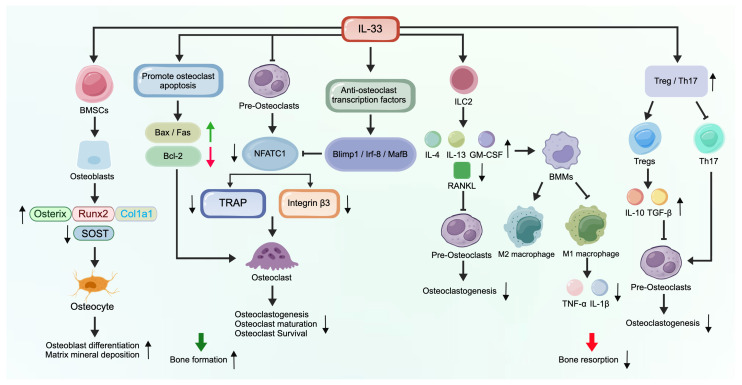
IL-33 regulates bone metabolism by acting on bone cells and immune cells.

These findings suggest that IL-33 exerts its bone-protective effects by regulating the functions of immune cells such as ILC2s, macrophages, and Tregs, thereby shaping an osteoimmune microenvironment that inhibits osteoclastogenesis and suppresses inflammation (Figure 3).

## 4. The Association Between IL-33 and Different Types of Osteoporosis

### 4.1. IL-33 and PMOP

Circulating IL-33 levels in PMOP remain clinically controversial. Ginaldi et al. [111] measured serum IL-33 levels in women with PMOP and healthy postmenopausal controls and found that serum IL-33 levels were significantly lower in the OP group. Correlation analysis further showed that serum IL-33 was significantly negatively correlated with the bone resorption marker C-terminal telopeptide of type I collagen (CTX) and positively correlated with the bone formation marker procollagen type I N-terminal propeptide (PINP). These findings suggest that IL-33 may exert a bone-protective effect and that its reduction may contribute to postmenopausal bone loss. In contrast, Ilesanmi-Oyelere et al. [112], in a cross-sectional study of postmenopausal women, reported significantly higher plasma IL-33 levels in the OP group than in the healthy control and osteopenia groups, and IL-33 levels were negatively correlated with bone mineral density (BMD). These discrepant findings may largely be attributable to methodological differences. First, different biological specimens were used (serum vs. plasma), and IL-33 is susceptible to the effects of coagulation and pre-analytical sample handling. Second, different assay platforms were employed. In the study by Ginaldi et al., IL-33 levels in the OP group were close to the lower detection limit of the ELISA kit, raising concerns regarding quantification reliability in the low-concentration range. Third, the study populations differed substantially. The former was a case–control study comparing clinically diagnosed osteoporotic patients with healthy controls, whereas the latter was a community-based cross-sectional stratified analysis of postmenopausal women, with a relatively small OP subgroup that may have been more vulnerable to individual inflammatory status and outlier effects. Fourth, adjustment for confounding factors was inconsistent, particularly with respect to inflammation-metabolic variables such as body mass index, C-reactive protein, ferritin, and PTH. Overall, these differences suggest that the association between circulating IL-33 and PMOP is highly sensitive to sample type, assay methodology, and population background. Future studies should compare serum and plasma IL-33 in parallel within the same cohort, standardize blood collection and storage procedures, use cross-validated high-sensitivity assay platforms, and apply multivariable adjustment for body mass index, C-reactive protein, ferritin, PTH, vitamin D, and bone turnover markers to more rigorously clarify the true relationship between IL-33 and PMOP.

Mechanistic studies by Macari et al. [113] have demonstrated that in their investigation of the regulatory mechanisms of estrogen receptor α (ERα) in alveolar bone metabolism, estrogen modulates IL-33 expression via the ERα signaling pathway. In ERα-knockout (ERα^−/−^) mice, local IL-33 mRNA expression levels in alveolar bone tissue were significantly elevated, accompanied by upregulation of pro-inflammatory factors such as TNF-α and IL-1β, suggesting that estrogen deficiency may indirectly affect bone metabolism by relieving the inhibition of IL-33. Macari et al. [114] further investigated the role of the IL-33/ST2 axis in estrogen-deprivation-induced bone loss. They found that in an ovariectomy (OVX) mouse model, ST2 deficiency prevented OVX-induced alveolar bone loss but had no significant effect on the femur, suggesting that the regulation of bone metabolism by the IL-33/ST2 axis under estrogen deficiency displays distinct site dependence. In addition, Cheng et al. [115] compared the therapeutic effects of extracorporeal shockwave therapy (ESWT), bisphosphonate (Aclasta), and human Wharton’s jelly-derived mesenchymal stem cells (WJMSCs) on OP in an OVX rat model. The results showed that all three treatments significantly improved BMD and cartilage integrity, whereas only ESWT and Aclasta significantly reduced IL-33 levels in serum and articular cartilage. These findings suggest that IL-33 may serve as a common target underlying the bone-protective effects of these therapies. Meanwhile, Keller et al. [116] first confirmed in vivo that IL-33 can directly inhibit osteoclastogenesis by generating osteoblast-specific IL-33-overexpressing transgenic mice (Col1a1-IL33). Under physiological conditions, these transgenic mice exhibited increased bone mass and significantly reduced osteoclast numbers compared with wild-type mice, whereas osteoblast numbers and bone formation parameters remained unchanged. This study provides direct in vivo evidence for IL-33 as a potential target against bone loss, including PMOP.

Taken together, unlike acute and infectious bone destruction, PMOP is essentially a non-infectious, chronic low-grade inflammatory state triggered by estrogen withdrawal. Under physiological conditions, endogenous IL-33 acts as a critical bone-protective barrier that suppresses osteoclastogenesis. However, under postmenopausal chronic inflammatory stress, the disruption of endogenous IL-33 homeostasis leads to the collapse of its physiological bone-protective function. Therefore, restoration of endogenous IL-33 homeostasis, rather than indiscriminate blockade or excessive supplementation, may represent one promising strategy worth investigating for PMOP treatment.

### 4.2. IL-33 and SOP

Immunosenescence refers to the progressive decline in immune function that occurs with aging. It is closely associated with a state of chronic low-grade inflammation, which is considered a major driver of age-related diseases such as SOP. In this context, Kaushal et al. [117] demonstrated in a D-galactose-induced aging mouse model, a widely used model of accelerated senescence, that IL-33 treatment alleviates inflammation by inhibiting Th17 cell differentiation and promoting Treg expansion. Meanwhile, IL-33 directly acts on osteoblasts to stimulate their differentiation and suppress cellular senescence, thereby significantly ameliorating bone loss and mitigating cognitive dysfunction. This study was the first to suggest the dual bone-protective and neuroprotective effects of IL-33 through regulation of the Th17/Treg balance under aging conditions. Furthermore, at the molecular level, De Martinis et al. [46] proposed the concept of the IL-33/IL-31 axis and noted that IL-33 and IL-31 exert opposing effects on bone metabolism. IL-33 exerts bone-protective effects by inhibiting RANKL-induced osteoclastogenesis and redirecting osteoclast precursor differentiation toward macrophages. In contrast, IL-31 promotes osteoclast differentiation via activation of the STAT1/3/5 and MAPK signaling pathways. Imbalance between these two cytokines during aging may exacerbate bone loss. According to the review by Aitella et al. [118], immunosenescence in older adults is accompanied by decreased sex hormone levels, elevated pro-inflammatory cytokines, and upregulation of bone metabolism-related factors (RANKL and DKK1). Dysregulation of the IL-33/IL-31 axis represents one of the key links connecting immunosenescence to bone metabolic disorders. 

Taken together, SOP is characterized by a chronic low-grade inflammatory microenvironment driven by immunosenescence, where IL-33 primarily functions as an endogenous anti-aging and bone-protective factor. However, age-related deterioration of the immune microenvironment disrupts the physiological balance of the IL-33/IL-31 axis and Th17/Treg ratio, thereby weakening its compensatory protective mechanisms. Consequently, correcting this immune imbalance within the aging microenvironment to restore the IL-33-driven protective signaling network represents a promising strategy for SOP intervention.

### 4.3. The Dual Role of IL-33 in Inflammatory Bone Loss

#### 4.3.1. RA

Elevated serum IL-33 levels have been reported in several studies of patients with RA [119,120]. Lee et al. [121] further demonstrated that plasma IL-33 levels were significantly higher in RA patients than in patients with osteoarthritis, and IL-33 mRNA and intracellular protein expression in synovial fibroblasts could be upregulated by proinflammatory factors (TNF-α, IL-1β) and polyinosinic-polycytidylic acid (poly I:C). These findings suggest that the local inflammatory microenvironment in RA joints can induce elevated IL-33 expression in synovial fibroblasts. Several studies have indicated that exogenous IL-33 aggravates RA symptoms by activating immune cells, promoting proinflammatory cytokine release, and enhancing bone destruction. Xu et al. [122] reported that exogenous IL-33 exacerbated synovial inflammation, cartilage damage, and severe bone erosion by activating MCs and promoting Th1/Th17 cell differentiation, whereas ST2 knockout attenuated these pathological changes. Kim et al. [96] further revealed that exogenous IL-33 activates MCs to release osteoclastogenic factors including TNF-α, IL-1β, IL-6, IL-17, and RANKL, and promotes monocyte differentiation into osteoclasts via cell–cell contact, thereby aggravating bone erosion. However, evidence also supports a bone-protective role of exogenous IL-33 in RA. Zaiss et al. [44] found in a TNF-α overexpression mouse model that exogenous IL-33 or an ST2-agonistic antibody significantly suppressed cartilage destruction and systemic bone loss. This study showed that IL-33 exerts bone-protective effects by promoting the secretion of anti-osteoclastogenic factors such as GM-CSF, IL-4, and IFN-γ, and directly redirecting osteoclast precursor differentiation toward AAMs. Notably, Li et al. [123] administered IL-33 neutralizing antibodies after disease onset in a collagen-induced arthritis mouse model. The results showed that anti-IL-33 antibody treatment significantly reduced clinical arthritis severity, decreased levels of proinflammatory cytokines (IFN-γ, IL-6, IL-12, IL-33, and TNF-α), and mitigated joint bone destruction. This study indicates that blocking endogenous IL-33 signaling may have therapeutic value during the inflammatory progression phase of RA.

In summary, IL-33 exhibits divergent functions in RA, likely reflecting differences in disease models, treatment routes, and microenvironmental contexts. Exogenous IL-33 may exacerbate inflammation and bone destruction by activating MCs and Th1/Th17 responses during early disease stages or in specific cell types. In contrast, under conditions of TNF-α-mediated systemic bone loss, exogenous IL-33 can exert anti-inflammatory and anti-osteoclastic protective effects. This dual nature suggests that therapeutic strategies targeting IL-33 must carefully consider disease stage, target cell populations, and local microenvironments to achieve precise intervention.

#### 4.3.2. Ankylosing Spondylitis (AS)/Psoriatic Arthritis (PsA)

Unlike RA, which is primarily characterized by bone erosion, AS is characterized by enthesitis and the resulting pathological bone formation. Hao et al. [43] reported that IL-33 may induce the differentiation of TREM2^+^ macrophages through STAT6 phosphorylation. These macrophages were shown to secrete CREG1, which may promote the osteogenic differentiation of ligament precursor cells via the IGF2R-PI3K-AKT axis, thereby contributing to pathological new bone formation. To our knowledge, this was the first study to directly link IL-33 to the characteristic new bone formation in AS. However, this proposed mechanism is currently based on a single primary study and awaits independent replication. In PsA patients, Shen et al. [124] found that elevated plasma levels of soluble ST2 (sST2, a soluble decoy receptor for IL-33) were associated with carotid plaque formation and impaired cortical bone microarchitecture, whereas IL-33 itself exhibited a low detection rate with no significant correlation. These findings suggest that the IL-33/ST2 axis may be involved in the comorbid processes of atherosclerosis and bone loss in PsA. Furthermore, Raimondo et al. [125] reported that stimulation of skin tissue with cytokines such as IL-33 induces the release of multiple pro-osteoclastogenic factors, including RANKL, which promote the differentiation of monocytes into osteoclasts. These findings suggest a potential pathogenic link between psoriatic skin inflammation and bone erosion in PsA. In this review, we refer to this proposed link as a “skin–bone axis,” by which skin-derived inflammatory mediators may facilitate osteoclastogenesis and thereby contribute to bone erosion. Concurrently, Li et al. [126] detected significantly elevated serum IL-33 levels in patients with PsA, yet found no significant correlation between IL-33 and disease activity or bone erosion, suggesting that IL-33 may mainly reflect systemic inflammatory status rather than directly drive joint destruction.

Collectively, the IL-33/ST2 axis appears to play distinct roles in AS and PsA. In AS, current evidence from a single study suggests that IL-33 may promote pathological new bone formation through the TREM2^+^ macrophage–CREG1 axis, representing a potentially abnormal tissue-repair response. In PsA, the IL-33/ST2 axis exerts a more complex role. IL-33 can induce the skin to release pro-osteoclastogenic mediators such as RANKL, thereby promoting bone erosion. However, circulating IL-33 levels are not significantly associated with bone erosion, whereas elevated sST2 is linked to impaired bone microarchitecture. These differences suggest that the function of the IL-33/ST2 axis varies across spondyloarthritis subtypes and is influenced by the local microenvironment, cellular targets, and disease stage. Further investigation is warranted to clarify its precise mechanisms in PsA and to validate the proposed pathway in AS.

#### 4.3.3. Periodontitis and Associated Bone Loss

Current research findings regarding the expression levels of IL-33 in local tissues from patients with periodontitis remain inconsistent. Lapérine et al. [127] and Malcolm et al. [128] both reported that IL-33 expression was significantly higher in gingival tissues from patients with chronic periodontitis than in healthy controls. However, Buduneli et al. [129] found that IL-33 concentration in gingival crevicular fluid (GCF) was significantly lower in patients with chronic periodontitis than in healthy controls. Papathanasiou et al. [130] reported that IL-33 was undetectable in all GCF samples examined. These discrepancies may be related to sample selection, disease severity, detection methods, and heterogeneity in sampling sites, suggesting that the expression of IL-33 in periodontitis may be influenced by multiple factors.

Functional studies have further revealed the protective role of endogenous IL-33/ST2 signaling in periodontitis. Velickovic et al. [95] found in an experimental model of periapical lesions that ST2-knockout mice exhibited more severe bone destruction, accompanied by increased Th1/Th17 cell infiltration and a higher proportion of RANKL^+^ T cells. Louisy et al. [131] further found that in a Porphyromonas gingivalis-induced experimental periodontitis model, female IL-33 knockout mice exhibited significantly exacerbated alveolar bone loss and an increased number of osteoclasts. Liu et al. [85] found in a modified ligature-induced periodontitis model that the absence of either IL-33 or ST2 led to exacerbated bone loss during the acute phase, accompanied by M1 polarization of macrophages and increased neutrophil infiltration. Collectively, these studies indicate that endogenous IL-33/ST2 signaling plays a crucial bone-protective role in periodontitis. In contrast to this endogenous protective effect, Malcolm et al. [128] found that exogenous IL-33 administration significantly exacerbated alveolar bone destruction in mice infected with Porphyromonas gingivalis. Mechanistic studies have shown that IL-33 treatment increased the proportion of T and B cells expressing RANKL in gingival tissue and draining lymph nodes, while administration of OPG completely blocked IL-33-induced bone destruction, suggesting that exogenous IL-33 exerts pro-osteoclastogenic effects by upregulating RANKL signaling. Addressing these contradictions, da Luz et al. [132] proposed that the role of IL-33 in periodontitis may depend on the stage and intensity of inflammation, as well as the local microenvironment. Wang et al. [47] further systematically elaborated on the dual nature of the IL-33/ST2 axis in oral diseases, noting that under physiological or mild mechanical stimulation, IL-33 can exert anti-osteoclastic and pro-repair effects. However, under acute or severe inflammatory conditions, IL-33, acting as an alarmin, may overactivate the immune response and exacerbate tissue destruction. This framework provides a plausible explanation for understanding the complex role of IL-33 in periodontitis.

In summary, endogenous IL-33/ST2 signaling exerts a bone-protective effect under steady-state conditions and during mild inflammation, whereas exogenous or excessive IL-33 may exacerbate bone destruction in the context of severe infection by inducing RANKL expression and promoting Th1/Th17 responses. This dual nature suggests that targeting the IL-33/ST2 axis as a therapy for periodontitis requires precise modulation of its activity to avoid the adverse effects associated with excessive activation or complete blockade.

#### 4.3.4. GIOP

Direct studies investigating the role of IL-33 in GIOP remain limited. The available evidence mainly suggests that IL-33 may indirectly participate in the development and progression of GIOP by regulating gut microbiota and systemic inflammation. Specifically, Guo et al. [133] conducted bidirectional mendelian randomization analysis combined with a clinical cohort study, and found that patients receiving glucocorticoid treatment exhibited significantly decreased abundance of Lachnospiraceae in the gut, accompanied by reduced levels of butyrate, its major metabolite. Meanwhile, serum levels of inflammatory factors including IL-33, IL-17A and TNF-α were elevated, and IL-33 levels were negatively correlated with Lachnospiraceae abundance. These findings indicate that glucocorticoids may indirectly increase the levels of inflammatory factors such as IL-33 by inducing gut dysbiosis and butyrate deficiency, thereby exacerbating bone loss. Nevertheless, this study was observational in nature, and changes in IL-33 served merely as a correlative marker rather than a direct therapeutic target.

Taken together, there remains a notable knowledge gap regarding the direct mechanistic role of IL-33 in GIOP. Current evidence only reveals potential indirect associations between IL-33 and GIOP. Further studies using specific animal models are needed to directly define the precise role of the IL-33/ST2 axis in glucocorticoid-induced bone loss.

#### 4.3.5. ONFH

Ma et al. [134] first reported significantly higher plasma IL-33 levels in patients with non-traumatic ONFH than in healthy controls. Further analyses revealed that IL-33 levels were correlated with CJFH classification (necrotic extent) and lateral pillar preservation, whereas no significant association was observed with ARCO stage. These findings suggest that IL-33 may be involved in the pathological process of ONFH and serve as a potential biomarker for prognostic evaluation. Zheng et al. [135] reported that serum IL-33 levels could distinguish FICAT stage II from stage III in patients with ONFH and were negatively correlated with CXCL12. These findings suggest that IL-33 may serve as a potential biomarker for early-to-midstage disease progression in ONFH. Some scholars believe that IL-33 may be released from necrotic bone cells or bone marrow cells and participate in the early inflammatory response of ONFH as an alarmin [136]. Li et al. [137] further elucidated the pathogenic mechanism of IL-33 in a glucocorticoid-induced ONFH mouse model. Exogenous IL-33 administration significantly aggravated osteonecrotic lesions in ONFH, as evidenced by an increased empty lacunar rate, expanded fibrotic area, and exacerbated trabecular bone destruction. IL-33 inhibited bone regeneration by suppressing osteoblast proliferation and differentiation, downregulating the expression of osteogenic marker genes including runt-related transcription factor 2 (Runx2), osteocalcin (OCN), and osteopontin (OPN), while upregulating RANKL to promote osteoclastogenesis. Blockade of IL-33 signaling using an anti-ST2L antibody reversed these effects, confirming a pathogenic role of the IL-33/ST2L axis in ONFH.

Regarding intervention strategies, Cheng et al. [138] found in a rat model of early-stage avascular necrosis of the femoral head that ESWT could downregulate the expression of IL-33 and IL-17A, upregulate ST2 levels, and thereby protect articular cartilage and improve bone structure. These findings suggest that ESWT may exert therapeutic effects by modulating the IL-33/ST2 axis. Hsu et al. [139] further confirmed that both ESWT and adipose-derived mesenchymal stem cells (ADSCs), as well as their combined therapy, significantly reduced the expression of pro-inflammatory factors such as IL-33, IL-5, IL-6, and IL-17A in the ONFH model, while simultaneously upregulating ST2 levels, thereby alleviating chondrocyte apoptosis and improving bone microstructure.

Taken together, in ONFH, IL-33 may serve not only as a biomarker reflecting disease severity but also as an alarmin involved in the early inflammatory response. It suppresses osteogenesis and promotes osteoclastogenesis, thereby accelerating bone destruction. Therefore, effectively downregulating aberrantly elevated IL-33 signaling may represent a key intervention strategy to delay the progression of ONFH (Table 1).

## 5. The Clinical Potential of IL-33 in Osteoporosis

### 5.1. The Value of Disease Monitoring

IL-33 levels appear to be associated with OP severity, fracture risk, and treatment efficacy. In OP, a study by Ginaldi et al. [111] demonstrated that the correlations between serum IL-33 levels and markers of bone turnover support its potential as a biomarker for monitoring dynamic changes in bone metabolism. In ONFH, Zheng et al. [135] found that serum IL-33 levels could distinguish FICAT stage II from stage III in patients, suggesting that IL-33 may serve as a potential biomarker for assessing disease progression. In RA, Xiangyang et al. [120] found that serum IL-33 levels in patients with RA were positively correlated with the modified sharp score, but showed no significant correlation with the disease activity score DAS28. Accordingly, IL-33 may serve as a potential biomarker for bone erosion. Furthermore, regarding treatment response, Cheng et al. [115] found in an OVX rat model that ESWT and aclasta treatment reduced IL-33 levels and improved BMD, suggesting that IL-33 may serve as a potential biomarker for evaluating therapeutic response.

### 5.2. The Value of Subtype Differentiation

Differences in IL-33 expression among distinct types of osteoporosis and related skeletal disorders may provide a basis for distinguishing between inflammation-related and non-inflammation-related osteoporosis. For example, IL-33 levels are significantly elevated in RA-associated bone loss [119,120], whereas they tend to decrease in PMOP [111], suggesting that IL-33 may exhibit opposite expression patterns in inflammatory and non-inflammatory bone loss. Furthermore, Talabot-Ayer et al. [119] found that serum and synovial fluid IL-33 levels were elevated in RA but undetectable in PsA, suggesting that differential IL-33 expression may be useful in differentiating RA from PsA. Meanwhile, IL-33 levels are elevated in ONFH [134], whereas changes in SOP appear more complex, suggesting that IL-33 expression may reflect differences in the pathological mechanisms of various bone diseases (Table 2).

### 5.3. Prospects for Therapies Targeting the IL-33/ST2 Axis

In various animal models of bone loss, exogenous IL-33 supplementation has demonstrated bone-protective effects. Zaiss et al. [44] found in a mouse model of inflammatory arthritis with human TNF-α overexpression that administration of recombinant IL-33 or an ST2 agonist antibody significantly reduced joint inflammation, inhibited cartilage destruction, and mitigated systemic bone loss. Kaushal et al. [117] further demonstrated in a D-galactose-induced aging mouse model that IL-33 treatment significantly attenuated bone loss while alleviating cognitive impairment. These studies suggest that IL-33 supplementation may have therapeutic potential for both inflammatory bone loss and age-related bone loss.

In addition to direct supplementation with IL-33, targeting its downstream immune cells and signaling pathways has also emerged as an important strategy for bone protection. Omata et al. [106] found in an arthritis model that ILC2s inhibited the secretion of inflammatory cytokines by macrophages via IL-4/IL-13, thereby indirectly mitigating bone destruction in arthritis. Transplantation or in vivo expansion of ILC2s reduced osteoclast numbers and increased bone volume, exerting bone-protective effects. Momiuchi et al. [108] further revealed that IL-33-activated ILC2s suppressed osteoclastogenesis by downregulating RANKL expression and secreting GM-CSF and IL-13 to drive polarization of monocytes/macrophages toward an M2 phenotype. Additionally, Zhang et al. [109] reported that IL-33/ST2 signaling promoted the expansion and function of reparative Tregs, which inhibited osteoclastogenesis through IL-4, TGF-β secretion, and direct cell–cell contact. These findings suggest that targeting downstream immune cells such as ILC2s and Tregs, or utilizing downstream cytokines including IL-4 and IL-13, may serve as alternative strategies to indirectly achieve bone protection.

Of note, Saleh et al. [99] reported that PTH significantly upregulated IL-33 mRNA expression in osteoblasts. Given that IL-33 exerts dual effects by suppressing osteoclastogenesis and promoting osteoblast mineralization, this suggests that the bone-forming action of PTH may be mediated, at least in part, by IL-33. Li et al. [140] reported preliminary preclinical evidence that Yishen Bugu Ye (YSBGY), a traditional Chinese herbal formula, increased serum IL-33 levels and reduced pro-inflammatory cytokines, including IL-1, IL-7, and TNF-α, in an osteoporotic mouse model. These findings suggest a possible association between YSBGY treatment and modulation of the IL-33-related inflammatory milieu. However, the underlying active constituents and mechanisms remain to be clarified. Cheng et al. [138] and Hsu et al. [139] demonstrated in rat models of ONFH that ESWT significantly decreased the expression levels of IL-33 and IL-17A while upregulating the receptor ST2, thereby protecting articular cartilage and improving bone structure. Cheng et al. [115] demonstrated in an OVX rat model that both ESWT and aclasta downregulated IL-33 levels while upregulating ST2 expression, and significantly improved BMD and preserved cartilage integrity. Qiu et al. [100] found that macrophages express IL-33 upon stimulation by black phosphorus, which directly acts on BMSCs to promote osteogenic differentiation, thereby facilitating bone regeneration. The above studies indicate that various existing therapeutic approaches may exert bone-protective effects by regulating the IL-33/ST2 axis.

## 6. Conclusions

The IL-33/ST2 axis represents a critical link between immune inflammation and bone remodeling, and its role in osteoporosis and related skeletal disorders is highly context-dependent. Current evidence indicates that IL-33 can exert osteoprotective effects by directly inhibiting osteoclast differentiation, enhancing type 2 immune responses, expanding Tregs, and promoting M2 macrophage polarization. However, under specific inflammatory conditions, IL-33 may also aggravate bone destruction by activating MCs, promoting Th17 differentiation, and upregulating RANKL signaling. Therefore, the biological effects of IL-33 are determined not by an intrinsically pro-inflammatory or anti-inflammatory nature, but rather by its cellular source, target cell type, local microenvironment, and the stage of disease.

A major challenge in this field is that most current studies still rely on global gene knockout models or exogenous intervention strategies, making it difficult to accurately distinguish the direct and indirect effects of IL-33 in different bone and immune cell populations. Future studies should integrate cell type-specific genetic models with single-cell sequencing, spatial transcriptomics, and dynamic imaging technologies to systematically dissect the spatiotemporal characteristics and functional switching of the IL-33/ST2 axis during inflammation initiation, bone loss progression, and tissue repair. Such efforts will be essential for clarifying whether IL-33 acts predominantly as a protective or pathogenic factor under distinct pathological conditions.

At the translational level, IL-33 and ST2 hold promise as candidate biomarkers for stratified management of bone diseases, although their clinical utility still requires further validation through standardized detection methods and large-scale prospective studies. A more practical future direction may be to incorporate the IL-33/ST2 axis into precision therapeutic frameworks and to explore its combination with anti-RANKL, anti-TNF-α, anti-IL-17, or bone anabolic agents according to inflammatory status, bone turnover characteristics, and disease subtype. Only after clarifying when to intervene, which cell populations to target, and which patients are most likely to benefit can the IL-33/ST2 axis be meaningfully translated from mechanistic research into clinical osteoimmunotherapy.

## Figures and Tables

**Table 1:** Expression, regulation and functions of the IL-33/ST2 axis in skeletal disorder.

Disease	IL-33/ST2 Axis	Molecular Markers	Cells	Function	Site	Species	References
PMOP	IL-33 ↓	CTX ↑ PINP ↓	-	Osteogenesis ↓	Femur	Human	[111]
	IL-33/ST2 ↑	TNF-α ↑ IL-1β ↑ RANKL/OPG ↑	Osteoblast ↓ Osteoclast ↑	Osteogenesis ↓	Maxillary alveolar bone	Mouse	[113]
	ST2^−/−^	TNF-α ↓ IL-10 ↑ Sema3A ↑	Osteoblast ↑	Osteogenesis ↑	Maxilla	Mouse	[114]
	IL-33 ↓ ST2 ↑	IL-31 ↑ BMP2 ↓	-	Osteogenesis ↑	Proximal femur, tibia, and spine	Rat	[115]
SOP	IL-33 ↑	IL-1β/TNF-α/IL-17 ↓, IL-10 ↑, p53/p21/pRB ↓, BACE1/p-tau ↓, CREB ↑, Runx-2/COL1/P1NP ↑, and CTX ↓	Th17 ↓, Treg ↑, and Osteoblast ↑	Osteogenesis ↑	Femur	Mouse	[117]
RA	IL-33 ↑	NF-κB ↓ IL-6/IL-8/MCP-1/MMP-1/3/13 ↓ RANKL ↑ IP-10 ↑	Osteoclast ↑	Osteogenesis ↓	Synovial tissue	Human	[121]
	IL-33/ST2 ↑	IL-17/IFNγ/TNF-α/IL-5/IL-12/IL-1β/IL-6/IL-13/GM-CSF/MCP-1/MIP-1α ↑	Mast cells ↑ Th1/Th17 ↑	Osteogenesis ↓	Synovial tissue	Human Mouse	[122]
	IL-33 ↑	TNF-α/IL-1β/IL-6/IL-17/RANKL/ MMP-9 ↑, TRAP/NFATc1 ↑	Mast cells ↑ Osteoclast ↑	Osteogenesis ↓	Synovial tissue	Human	[96]
	IL-33/ST2 ↑	TRAP/NFATc1 ↓, IL-4/IFN-γ/GM-CSF ↑, Cathepsin K ↓	Osteoclast ↓ Alternatively activated macrophages ↑	Osteogenesis ↑	Tibia	Mouse	[44]
	IL-33 ↓	IFN-γ/IL-6/IL-12/ TNF-α ↓	-	Osteogenesis ↑	Knee joints	Mouse	[123]
AS	IL-33/ST2 ↑	p-STAT6 ↑, IL-4/IL-13 ↑, CREG1 ↑	TREM2^+^ macrophages ↑ Ligament-derived progenitor cells ↑	Osteogenesis ↑	Spinal ligament, Hind paws	Human Mouse	[43]
PsA	IL-33 ↑	RANKL/TNF-α/IL-6/ OPN ↑, MCP-1/MIP-1α/β, RANTES/IP-10/ MIG ↑,	Osteoclast ↑	Osteogenesis ↓	Skin	Human	[125]
Periodontitis	IL-33 ↑	RANKL ↑	Osteoclast ↑	Osteogenesis ↓	Gingival tissue, Alveolar bone	Human Mouse	[127]
	IL-33/ST2 ↑	RANKL ↑	T/B cells ↑	Osteogenesis ↓	Gingival tissue, Alveolar bone	Human Mouse	[128]
	IL-33/ST2 ↓	TNF-α/IL-6/IFN-γ/ IL-17 ↑, RANKL ↑, OPG ↓	Th1/Th17 ↑, Dendritic cell ↑, Osteoclast ↑	Osteogenesis ↓	Periapical tissue, Mandible	Mouse	[95]
	IL-33^−/−^	-	Osteoclast ↑	Osteogenesis ↓	Maxilla, Femur fifth, Lumbar vertebra	Mouse	[131]
	IL-33^−/−^ ST2^−/−^	IL-6 ↑, RANKL ↑	M1/M2 macrophages ↑, Neutrophils ↑, Osteoclast ↑	Osteogenesis ↓	Gingival tissue, Peri-root tissue, Alveolar bone	Mouse	[85]
GIOP	IL-33 ↑	-	-	Osteogenesis ↓	Femoral head	Human Rat	[133]
ONFH	IL-33/ST2 ↑	Runx2/OCN/OPN/ COL1/ALP ↓, RANKL ↑, IL-1β/IL-6/IL-4 ↓	Osteoblast ↓ Osteoclast ↑	Osteogenesis ↓	Femoral head	Mouse	[137]

↑: Increase; ↓: Decrease.

**Table 2:** Summary of IL-33 and sST2 expression patterns and their clinical correlations across osteoporosis and related skeletal disorder.

Disease	Age (Year)	Sample Size (*n*)	Sample	Method	IL-33 Level (pg/mL)	sST2 Level	References
PMOP	Patients: 65.42 ± 9.59 Control: 62.07 ± 8.34	Patients: 50 Control: 28	Serum	ELISA (USCN Life Science Inc., Houston, TX, USA)	Patients: 3.53 ± 2.45 Control: 13.72 ± 5.39 (*p* = 0.009), IL-33 vs. PTH (r = 0.314, *p* = 0.026), IL-33 vs. P1NP (r = 0.373, *p* = 0.011), IL-33 vs. CTX (r = −0.455, *p* = 0.002)	Not measured	[111]
PMOP	63.2 ± 4.6	Osteoporotic: 13 Osteopenic: 34 Healthy: 39	Plasma	LEGENDplex Multi-Analyte Flow Assay kit (BioLegend, Inc., San Diego, CA, USA) and Gallios flow cytometer (Beckman Coulter, Inc., Brea, CA, USA)	Osteoporotic: 13.88 ± 37.44 Osteopenic: 0.79 ± 3.32 Healthy: 2.83 ± 11.19 (F-value = 3.147, *p* = 0.048)	Not measured	[112]
RA	RA: 56; 27–80 ^a^ OA: 60; 34–77 ^a^ PsA: 59; 35–83 ^a^	RA: 11 OA: 9 PsA: 9	Serum	ELISA (R&D Systems, Abingdon, UK)	RA vs. OA: Up (*p* = ns), RA vs. PsA: Up (*p* < 0.05)	sST2: RA vs. OA: Up (*p* < 0.05), RA vs. PsA: Up ( *p* = ns)	[119]
	RA: 51 ± 17 Control: 47 ± 14	RA: 121 Control: 47	Serum	ELISA (R&D Systems, Minneapolis, MN, USA)	RA: 138.6 ± 16.5 Control: 42.8 ± 3.0 (*p* = 0.0004), IL-33 vs. RF (r = 0.39, *p* = 0.001), IL-33 vs. MMP-3 (r = 0.23, *p* = 0.01), IL-33 vs. Modified Sharp Score (r = 0.53, *p* = 0.0001)	Not measured	[120]
	RA: 51.7 ± 11.2 OA: 63.6 ± 9.7	RA: 30 OA: 30	Serum	ELISA (R&D Systems, Minneapolis, MN, USA)	RA: 62.34 (0, 1804) OA: 0 (0, 94.84) ^b^ (*p* = 0.001)	Not measured	[121]
PsA	Carotid plaque present: 57.2 ± 9.3 Carotid plaque absent: 50.1 ± 9.7	Carotid plaque present: 33 Carotid plaque absent: 47	Plasma	ELISA (R&D Systems, Minneapolis, MN, USA)	Not reported	Carotid plaque present: 11.2 ± 4.5 ng/mL Carotid plaque absent: 7.7 ± 3.7 ng/mL	[124]
ONFH	ONFH: 49.2 ± 12.4 Control: 49.6 ± 16.0	ONFH: 40 Control: 40	Serum	ELISA (RayBiotech, Inc., Atlanta, GA, USA)	ONFH: 11.48 ± 8.34 Control: 5.30 ± 4.36 (*p* < 0.001), IL-33 vs. Disease history (r = −0.136, *p* = 0.403)	Not measured	[134]

^a^ Age: Mean; range. ^b^ Numbers are shown as the median (min, max).

## Data Availability

No new data were created or analyzed in this study.
